# Identification of Distant Regulatory Elements Using Expression Quantitative Trait Loci Mapping for Heat-Responsive Genes in Oysters

**DOI:** 10.3390/genes12071040

**Published:** 2021-07-05

**Authors:** Kexin Zhang, Jinpeng Wang, Fangfang Ding, Ruihui Shi, Wei Wang, Guofan Zhang, Li Li

**Affiliations:** 1 CAS and Shandong Province Key Laboratory of Experimental Marine Biology, Center for Ocean Mega-Science, Institute of Oceanology, Chinese Academy of Sciences, Qingdao 266071, China; 17854203920@163.com (K.Z.); wangjinpeng0225@163.com (J.W.); qd_dingfangfang@163.com (F.D.); shiruihui123@163.com (R.S.); wangwei@qdio.ac.cn (W.W.); gfzhang@ms.qdio.ac.cn (G.Z.); 2 Laboratory for Marine Biology and Biotechnology, Pilot National Laboratory for Marine Science and Technology, Qingdao 266237, China; 3 University of Chinese Academy of Sciences, Beijing 100049, China; 4 Institute of Animal Science and Veterinary Medicine, Shandong Academy of Agricultural Sciences, Jinan 250100, China; 5 Laboratory for Marine Fisheries Science and Food Production Processes, Pilot National Laboratory for Marine Science and Technology, Qingdao 266237, China; 6 National and Local Joint Engineering Laboratory of Ecological Mariculture, Qingdao 266071, China

**Keywords:** oyster, thermal adaption, heat-responsive gene, expression quantitative trait loci (eQTL) mapping, distant regulatory locus

## Abstract

Many marine ectotherms, especially those inhabiting highly variable intertidal zones, develop high phenotypic plasticity in response to rapid climate change by modulating gene expression levels. Herein, we examined the regulatory architecture of heat-responsive gene expression plasticity in oysters using expression quantitative trait loci (eQTL) analysis. Using a backcross family of *Crassostrea gigas* and its sister species *Crassostrea angulata* under acute stress, 56 distant regulatory regions accounting for 6–26.6% of the gene expression variation were identified for 19 heat-responsive genes. In total, 831 genes and 164 single nucleotide polymorphisms (SNPs) that could potentially regulate expression of the target genes were screened in the eQTL region. The association between three SNPs and the corresponding target genes was verified in an independent family. Specifically, Marker13973 was identified for heat shock protein (HSP) family A member 9 (*HspA9*). Ribosomal protein L10a (*RPL10A*) was detected approximately 2 kb downstream of the distant regulatory SNP. Further, Marker14346-48 and Marker14346-85 were in complete linkage disequilibrium and identified for autophagy-related gene 7 (*ATG7*). Nuclear respiratory factor 1 (*NRF1*) was detected approximately 3 kb upstream of the two SNPs. These results suggested regulatory relationships between *RPL10A* and *HSPA9* and between *NRF1* and *ATG7*. Our findings indicate that distant regulatory mutations play an important role in the regulation of gene expression plasticity by altering upstream regulatory factors in response to heat stress. The identified eQTLs provide candidate biomarkers for predicting the persistence of oysters under future climate change scenarios.

## 1. Introduction

Climate change has resulted in rapid global ocean warming [1], which threatens the survival and performance of marine ectotherms [2]. Studies suggest that phenotypic plasticity, the ability to exhibit several different phenotypes with the same genotype [3], plays an important role in species’ responses to global warming [4,5,6,7]. Oysters have high phenotypic plasticity, which at morphological and molecular levels allow them to adapt to highly variable intertidal zones. This ability makes them ideal representatives of intertidal organisms for the study of adaptive mechanisms in response to heat and other environmental stresses [8,9,10]. Gene expression variation accounts for much of the phenotypic variation among populations and individuals in response to rapid climate change [11,12,13,14].

Energy balance and cellular homeostasis are fundamental for survival and adaption to environmental stress [15]. Transcriptomic and proteomic analyses reveal changes in genes and proteins that are involved in cellular homeostasis, protein stability, metabolic adjustment, signaling transduction, and ion transportation and so on under heat stress in marine organisms. Heat shock proteins (HSP)s and genes related to metabolic adjustment and ion transport show differential expression in fish exposed to elevated temperatures [16,17]. In shrimp, HSPs are strongly upregulated under heat stress [18,19]. Genes related to protein homeostasis, oxidative stress mediation, osmolyte transport, and apoptosis are expressed differentially under different temperatures in shellfish [10,20,21,22,23,24,25]. Elucidating the genetic basis of heat-responsive gene expression will reveal the underlying mechanisms of phenotypic plasticity and lead to a better understanding of thermal adaption to global warming.

Expression quantitative trait locus (eQTL) mapping, which locates genetic loci related to mRNA transcript abundance in a segregation population [26,27], can link genetic variants to gene expression and phenotypes [11]. eQTLs are genomic regions containing several genetic variants that can influence gene expression [11] and are classified into local and distant eQTLs according to their distance from the target gene [28]. Most local eQTLs regulate gene expression through cis-acting regulation, which directly influences target gene expression and protein characteristics (cis-eQTLs). Distant eQTLs are usually trans-acting [11], and can change target gene expression by influencing the function, structure, and expression of other factors (trans-eQTLs). However, distant cis and local trans effects have also been reported [28]. Genome-wide eQTL mapping for the detection of genetic loci linked to phenotypes has been used for many species [29] to elucidate the underlying mechanisms of complex traits [30,31,32,33,34,35]. Aquatic ectotherms, the most susceptible groups in response to the deterioration of the environment, are ideal organisms to study to unravel the underlying mechanisms of high plasticity in gene expression. However, the detection of genetic variants of environment-responsive gene expression by eQTL mapping in marine ectotherms has only been studied thus far in three-spined stickleback [36].

Oysters have high economic value and are top ranking in global mariculture but unfortunately suffer summer mortality [37,38,39] mainly due to high temperatures [40]. Therefore, understanding the mechanism of high thermal adaption in oysters will elucidate prediction of marine organisms’ adaptive potential to global warming and help tackle oyster summer mortality. Moreover, oysters have high fecundity, which facilitates eQTL analysis. The Pacific oyster, *C. gigas*, and its sister species, *C. angulata*, are distributed along the northern and southern coasts of China, respectively [41]. The two species can interbreed, and both exhibit thermal tolerance divergence at physiological and molecular levels [8]. The basic and plastic expression of heat-responsive genes is differentiated between the two species [10].

This study aimed to elucidate the genetic variants regulating heat-responsive gene expression under acute heat stress by eQTL mapping. We identified several eQTLs using a previously reported high-density genetic map [42]. We further validated the association between the single nucleotide polymorphisms (SNPs) located in the eQTLs and the expression of the corresponding heat-responsive genes in an independent family. To our knowledge, this is the first study to identify the potential regulatory locus of environmentally responsive genes by integrating differential gene expression into QTL mapping in mollusks. Our results will help to highlight the adaptive mechanisms of marine organisms in response to environmental stress and provide a potential target region of genetic modification in the selection for thermal resistance in oysters.

## 2. Materials and Methods

### 2.1. Origin and Background of Oysters

Two backcross families of *C. gigas* and *C. angulata* were used in this study. ZF2-3 included 106 individuals and was used for eQTL mapping. ZF was used for subsequent association analysis between genotypes and gene expression levels. The two families were produced in a hatchery in Qingdao, China (36°12′ N, 120°41′ E) in 2012. Both the male parents of the two backcross families were the progeny of the hybrid family of *C. gigas* and *C. angulata*, which were produced and cultured in Nan’ao Island, Shantou, China (23°25′ N, 117°01′ E). Both the female parents were 2-year-old wild *C. gigas* from Jiaonan, Qingdao, China (35°44′ N, 119°56′ E). The procedure to construct the families were described in the previous study [42]. Briefly, both the parent oysters were dissected manually, the gonads of male parents were rinsed in seawater for activation of sperm for 30 min, and the gonads of female parents were rinsed in seawater for activation of egg for 1 h. Few sperm add to eggs for fertilization (no more than 10 sperm around one egg). The oyster larval were reared in a 70 L tank for 1 month until they attached to the settlement substrate. And then they were reared in a marine farm in Qingdao.

### 2.2. Heat Stress Simulation

Before the heat stress simulation, the progeny was collected at 14 months-old from the marine farm and transferred to the indoor laboratory, where they were reared in natural seawater and feed with culture algae for two weeks. For the acute heat stress treatment, seawater was heated and maintained at 35 °C using an automatic temperature controller connecting with a heating rod (ZHONGKEHAI, China), Both the progeny of ZF2-3 and ZF families was placed into the seawater at 35 °C for 3 h. Air was pumped into the water throughout the entire experiment.

### 2.3. DNA and RNA Extraction

Gills and mantles were sampled from each heat-treated oyster, immediately frozen in liquid nitrogen, and stored at −80 °C for RNA and DNA extraction, respectively. Approximately 20 mg of gill tissue was used for total RNA extraction, according to RNAprep pure Tissue Kit (TIANGEN, Beijing, China) instructions. Total DNA was extracted using a TIANamp Marine Animals DNA Kit from approximately 30 mg of mantle tissue. The DNA and RNA concentrations were measured using a NanoDrop 2000 spectrophotometer (Thermo Fisher Scientific, Waltham, MA, USA). Sample quality was identified using 1.0% agarose gel electrophoresis.

### 2.4. Genotyping-by-Sequencing

For ZF2-3 family, we used the same SNP dataset produced by genotyping-by-sequencing as described in Wang et al. [42]. Briefly, the EcoRI-HinfI combination was selected for digesting the genomic DNA; the digested DNA fragments were ligated to adaptors with barcodes; barcoded DNAs of parents and progenies of ZF2-3 family were mixed together and separated on a 2% agarose gel; DNA fragments of 400–600 bp (including adaptor) were selected and purified; the selected DNA fragments were amplified by PCR; PCR products were purified with Agencourt AMPure XP beads (Beckman Coulter, High Wycombe, UK); purified DNA fragments were sequenced on IlluminaHiseq 2500 platform (Illumina, San Diego, CA, USA). The Stacks software was used to analyze the sequencing data and to genotype DNA variations in the mapping family. The Pacific oyster reference genome oyster_v9 (GenBank accession no. GCA_000297895.1) was applied for SNP calling [25].

### 2.5. Detection of Thermal Responsive Gene Expressions

Twenty-one previously-reported thermal responsive candidate genes [10,24,25], were selected for quantitative real-time PCR. Gene IDs and primers are shown in Table 1. All primer design was conducted with Primer Premier 5. Elongation factor 1 *α* (*EF1α*) was used as the reference gene. One microgram of RNA from each sample was used for cDNA synthesis with the *Evo M-MLV* RT Kit II (AG, China). The synthetic cDNA was diluted 20 times for subsequent RT-PCR. The reaction system was: 2 μL cDNA, 10 μL SYBR Green 2X Supermix (TaKaRa Bio Inc., Shiga, Japan), 6.8 μL DEPC H_2_O, 0.4 μL sense and anti-sense primer, and 0.4 μL ROX Dye II. The reaction program was as follows: 30 s at 95 °C, 5 s at 95 °C for 40 cycles, and 30 s at 60 °C. The melt curve program was as follows: 15 s at 95 °C, 1 min at 60 °C, 30 s at 95 °C, and 15 s at 60 °C. RT-PCR was conducted using the ABI7500 Fast Real-Time Detection System (Applied Biosystems, Foster City, CA, USA). Relative gene expression was determined using the 2^−^^ΔΔCt^ method [10,43].

### 2.6. eQTL Analysis

The aforementioned relative gene expression was used as the phenotype for eQTL mapping, and genotype data were acquired from a high-density SNP genetic linkage map constructed by the aforementioned backcross family [42]. The construction process was described in detail by Wang et al. [42]. QTL mapping analysis was conducted to detect eQTLs for corresponding gene expression regulation using MapQTL6 [44], interval mapping (IM), and restricted multiple QTL model mapping (rMQM). An IM model was used for the selection of cofactors. eQTLs were acquired using rMQM with multiple selections of cofactors. A permutation test was conducted 3000 times at *p* < 0.05 to determine the significant LOD threshold. eQTLs with LOD scores exceeding the chromosome-wide LOD threshold were considered significantly related to gene expression. Candidate genes were screened within a 100 kb region around these significant SNP markers in eQTL regions. Enrichment and classification analysis of Gene Ontology (GO) [45] and Kyoto Encyclopedia of Genes and Genomes (KEGG) pathways [46] were performed with R. Fisher’s exact test was used for the GO enrichment analysis. GO enrichment results were ranked by *p*-value and the top 20 were selected to visualize the results.

### 2.7. Verification of eQTLs for an Independent Family

The relationship between each marker genotype and expression of the corresponding gene was investigated using the Mann-Whitney test, and the threshold for a significant difference was set at 0.05.

#### 2.7.1. Genotyping by SNaPshot

SNPs in the eQTL regions that had no mutations at 30 bp upstream and downstream were selected for association analysis. Genotyping was conducted using SNaPshot (3730xL × L DNA Applied Biosystems) in the ZF hybrid groups. Peripheral amplification primers and unidirectional oligonucleotide primers of different lengths were designed for these mutations (Table S1). After the multiplex reaction, the PCR product was separated by electrophoresis with five-color fluorescence detection and analyzed using Gene Mapper 4.1. Multiple SNP sites were detected simultaneously.

#### 2.7.2. Statistical Analysis

Allele frequency and genotype frequency were calculated using SHesis (http://analysis.bio-x.cn/myAnalysis.php, accessed on 11 August 2020) [47,48,49]. Since our data didn’t meet the normal distribution and homogeneity of variance, non-parametric test is adopted in our analysis. Specially, Mann-Whitney test was used for the analysis of variance in gene expression levels among different genotypes with two genotypes. For three genotypes, Kruskal-Wallis test was used. All analyses were performed using GraphPad Prism software. *p* < 0.05 was considered statistically significant.

## 3. Results

### 3.1. Gene Expression Analysis

The gene expression data are shown in Supplementary Table S2. The gene expression exhibited large phenotypic fold changes (8–642) and the distribution was positively skewed (Supplementary Figure S1). Phenotypic variation was rich, and the variable coefficient of each gene expression level was greater than 0.5.

### 3.2. eQTL Mapping and Distant eQTLs for Candidate Gene Expression

A total of 56 significant eQTLs were mapped for 19 of the 21 genes, covering 164 SNPs. The phenotypic variation explanation (PVE) ranged from 6% to 26.6%. eQTL details and mapping results are shown in Table 2 and Supplementary Figure S2, respectively. All the investigated genes and their related eQTLs (distant) were located on different scaffolds. Most SNPs affected the expression of only one target gene, whereas 29 SNPs affected two target genes. Three important SNPs affected the expression of more than two heat-responsive genes. Marker7064 was located in eQTL regions of three target genes: *HSPA12A*, *HSPA13* and *HSPA12B-12492*. Four genes (*HSPA12A*, *HYOU1*, *HSPA13* and *HSPA12B-12492*) colocalized with the same regulatory SNP: Marker25714. Marker13973 also was detected for *GAK*, *HSPA4*, *HSPA9*, and *HSPB1*.

A total of 831 candidate genes around these 164 SNPs are shown in supplementary Table S3. These genes were enriched in 93 KEGG pathways that mainly participated in metabolism, genetic information processing, environmental information processing, cellular processes, and human diseases (Figure S3). Functional enrichment analysis suggested that candidate genes were mainly involved in protein heterodimerization activity, DNA binding, nucleosome assembly, and centrosome duplication (Figure S4). Furthermore, 10 transcription factors were detected in the eQTLs of five target genes (*HSPA5-08834*, *HYOU1*, *HSPA13*, *ATG7*, *RNF123*).

### 3.3. Verification of Distant eQTLs in Different Families

Eleven critical distant regulatory SNPs located in nine distant regulatory regions for seven genes were genotyped and tested for associations with the expression of the corresponding genes in family ZF; Marker28123 and Marker30775 for *HSPA13*, Marker12828 and Marker11127 for *HSPA5-15492*, Marker13973 for *HSPA4*, *HSPA9*, and *HSPB1*, Marker61915 for *HSPA9*, Marker35461 for *BRKS2*, Marker20478 for *CLCN2*, Marker14346-48 and Marker14346-85 for *ATG7*, and Marker10740 for *HSPA5-08834*. Only six distant-acting loci (Marker 28123, Marker13973, Marker35461, Marker20478, Marker14346-48, and Marker14346-85) for seven genes were found to be polymorphic in the verification family. The genotype and allele frequencies of these six SNPs are shown in Supplementary Table S4.

**Figure 1:**
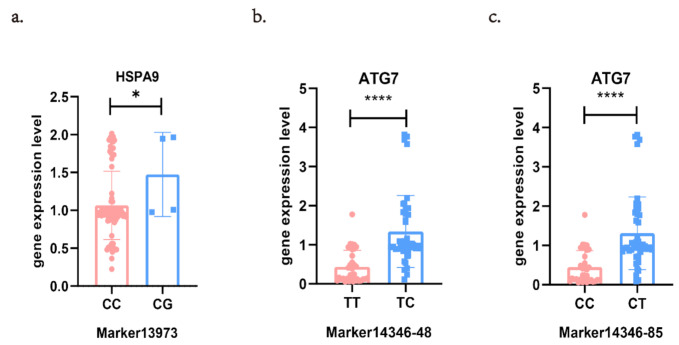
Association between different genotypes of single nucleotide polymorphisms (SNPs) and the gene expression level. (**a**) *HSPA9* gene expression level with different genotypes of Marker13973. (**b**) *ATG7* gene expression level with different genotypes of Marker14346-48. (**c**) *ATG7* gene expression level with different genotypes of Marker14346-85. The error bars denote the standard error of the mean. Asterisks indicate significant differences between different genotypes of SNPs. * indicates *p* < 0.05. **** indicates *p* < 0.0001.

The association between Marker13973 and the expression level of *HSPA9* was significant (*p* < 0.05) using the Mann-Whitney test (*p* = 0.0395, *n* = 85, Figure 1a, Table S5). Gene expression analysis indicated that individuals with the CG genotype had higher expression levels. Both Marker14346-85 and Marker14346-48 were significantly associated with the expression level of *ATG7* (*p* < 0.0001, *n* = 78, Figure 1b,c, Table S5). Individuals with CT at Marker14346-85 and TC at Marker14346-48 showed higher *ATG7* expression levels. Marker14346-85 and Marker14346-48 were 36 bp apart and in complete linkage disequilibrium (D’ = 1). Three haplotypes were identified: CT, CC, and TT (*p* < 0.05, Table S6).

## 4. Discussion

Herein, we detected 56 distant eQTLs, including 164 SNPs, without a local regulatory locus. The phenotypic variation of single regulatory loci at the transcript level was generally higher (6–26.6%) than growth-related traits (0.6–13.8%) using the same genetic map in Pacific oysters [42], which may due to the complexity of growth-related traits. However, the PVE in our study was generally lower than 8.4–59.7% of the tuber starch content relative genes at the transcript level in potatoes [50]. Also, a major local regulatory locus could even explain 70% of the muscle *IGF2* expression variance in pigs [51]. The limitation of the number and density of markers in the linkage map may explain why we did not detect any major local-eQTL in our study.

This study contains all distant changes and no local changes, which corroborate other studies’ findings of more distant than local changes [31,33,52,53]. A gene can be regulated by many distant mutation loci, but only a few local loci, which may be the reason why a higher proportion of distant eQTL has been reported [54]. Many studies considered that local eQTLs played a stronger and more significant role in regulatory ability than distant eQTLs because of their local activity [55,56]. However, recent studies have also realized the importance of distant eQTLs [31,57,58], which account for the majority of heritability in human gene expression and participate in gene-environment interactions [11]. Furthermore, one distant eQTL in a hotspot region may influence many genes by working in gene networks.

We also found three distant regulatory SNPs linked with three or four heat-responsive genes, which may be located in potential distant eQTL hotspot regions. Regulators such as transcription factors, nuclear receptors, miRNAs and small nuclear RNAs usually cluster near distant eQTL hotspot regions [31]. We found one regulatory element in our potential distant eQTL hotspot regions, *RPL10A*.

**Figure 2:**
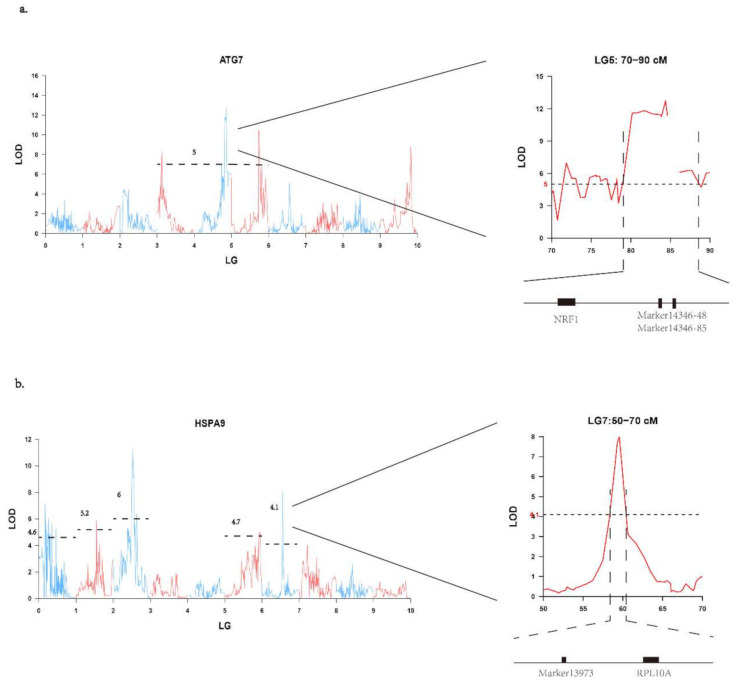
Plots of expression quantitative trait loci (eQTL) mapping and candidate regulatory loci and genes *HSPA9* and *ATG7*. (**a**) eQTL mapping of *HSPA9*. (**b**) eQTL mapping of *ATG7*. The black horizontal dashed line on each plot represents the chromosome-wide threshold of significance. Black rectangles represent candidate genes. Black vertical lines represent the regulatory single nucleotide polymorphism (SNP).

In this study, candidate genes within distant regulatory regions were enriched in pathways of environmental and genetic information processing. Other studies have illuminated that distant eQTLs can cause the functional divergence of several components that transduce environmental signals into gene expression changes [59]. Our results further complement the viewpoint that distant regulatory loci might influence the expression or function of regulatory elements that respond to environmental changes. In addition to linkage analysis, the associations between one distant regulatory SNP and *HSPA9* and between two distant regulatory SNPs and *ATG7* were further verified in an independent population, implying that these SNPs are important distant regulatory loci. The distant regulatory SNPs could affect the expression and function of target genes by mediating other regulatory factors. ATG (autophagy-related) proteins are the core of the autophagic machinery. *ATG7* encodes an E1-like activating enzyme and facilitates autophagic vesicle expansion by working with microtubule-associated protein light chain 3 (*LC3*) and *ATG12* [60,61]. In the distant eQTL regions of *ATG7*, one transcription factor, nuclear respiratory factor 1 (*NRF1*) was detected 3 kb upstream from the two distant regulatory SNPs (Figure 2a). *NRF1* encodes a transcription factor that activates the expression of metabolic genes involved in cellular growth and development [61]. In combination with the finding that *NRF1* can upregulate the expression level of *ATG7* by binding to its promoter in humans [62], we propose that the two distant regulatory SNPs (Marker14346-48 and Marker14346-85) might regulate the expression level of *ATG7* by affecting the expression of *NRF1*. The eQTLs and the validated SNPs provide candidate regions and markers to predict the adaptive potential in response to climate change. HSPAs are ubiquitous molecular chaperones that participate in many biological processes [63,64,65]. In the distant eQTL regions of HSPAs, we found *RPL10A*, a highly conserved gene with a variable number of spliceosomal introns in most organisms [66], approximately 2 kb downstream from the regulatory SNP of *HSPA9* (Figure 2b). We suggest that this distant regulatory locus Marker13973 might influence the expression of *HSPA9* by regulating the synthesis of *RPL10A*. The regulatory relationship between *RPL10A* and *HSPA9* was already elaborated in white shrimp [67].

Our results demonstrate the importance of distant eQTLs in adaptive evolution and elucidate the underlying regulatory mechanism of thermal adaption. The three regulatory SNPs identified might be used as potential regulatory loci of thermal adaption and could be applied to genetic improvements of heat-resistant oysters. However, we did not confirm the regulatory relationship among SNPs, gene expression, and high temperature resistance traits, which could be topics of further study.

## Figures and Tables

**Table 1:** Heat responsive Gene ID and primer, F represent sense primer, R represent anti-sense primer.

Gene Name	Gene ID	Primer Sequence (5′–3′)
HSPA4	CGI_10017255	F:ACCAAGCACAACTCTGAAT
		R:AGGCTGAGTATCCACAATG
HSPA5-08834	CGI_10008834	F:CCAGAACGACAACAACAGACTCTCA
		R:TTGGCTTCAACCTTCTCCTTCACTT
HSPA12A	CGI_10002491	F:CCACGTCATCAGGTCGATACAGAGA
		R:GCAATATGCTGTCTACGGCTGGTT
HSPA12B-22078	CGI_10022078	F:CTCTCACTGCGGACAAACTTCGT
		R:GCTTTCCACTTCTGGGTGCTGTAA
PARP4	CGI_10013249	F:ATAGCCAGGGTTGGAGCAGGTT
		R:ACGGATACCGAGGTCAGCACTG
HYOU1	CGI_10027129	F:CTAACGGACGAGGACCACGAGAA
		R:ACCTCCTTCTTGGCATCCTCTTCC
HSPA13	CGI_10027222	F:TCCATACCAAGCCTGAGCTGAAGA
		R:TACACGGAGTAGCGACACATCCA
HSPA12B-12492	CGI_10012492	F:CTGAAGATCCAGGACTTGCTGTGTT
		R:GCCAGCTCTGAATGCCATAAGTGT
HSPA9	CGI_10016162	F:TCACTCCGCTGTCGTTGGGTAT
		R:TGTCCATCAGCAGCCGTTGAGA
HSPA5-15492	CGI_10015492	F:ACCGTGGAGTCAACCCTGATGA
		R:CGTCCAACAGCAGAAGGTCACC
HSP68	CGI_10002594	F:TCAGCCAAGGACAAGAGCACAG
		R:TCGGCCTCGTTCACCATTCTCT
SIP1	CGI_10004164	F:CGCCATTACGGACGGCAAGAA
		R:ACGGTAATGTGGTCAGGCTCGA
HSPB1	CGI_10017582	F:CCGAAGGAAGAGGACCAGGAGATG
		R:CGAACACCGACAGGTCTAAACTCTC
DJB13	CGI_10006977	F:CACATTTCCAGAAGAAGGCGACCA
		R:GAACCTTGGCAGTGTGGATCAGATT
DNJB4	CGI_10009495	F:GGAGGAGACGACCCGTTTGCTA
		R:GTTGCCCGCCGAAATGGAACA
CLCN2	CGI_10022926	F:CCTGGTTACATTGGTGATTTCC
		R:CTCAGCCTGTCCTGTCGTC
BRKS2	CGI_10010977	F:TGGATAGTCACGGATTAGTAGAG
		R:GGGGTTCCTGAGTTTTCG
MTMR8	CGI_10006555	F:TGTTTGTTCCTGCCTCCG
		R:CCTTTACCAGCCGCCTTA
ATG7	CGI_10025698	F:CACTGGACACCCCTGAAC
		R:GCAACAAAGAAACCCTGAT
GAK	CGI_10028689	F:CGACAAAGGACAGTGCGAGTA
		R:CTGAATGGCTGACCAAGACC
RNF123	CGI_10004787	F:TCCGTCATCTCCCACTCC
		R:TTCTTTGTCCTTTCTTCCCT

**Table 2:** Summary of candidate expression quantitative trait loci (eQTL) regions of 19 genes by eQTL mapping. eQTL names, linkage groups, confidence intervals (CIs), nearest markers, peak of LODs, number of markers, and phenotypic variation explanation (PVE) are shown in the table.

Gene ID	eQTL Name	Linkage Group	CI (cM)	Nearest Marker	Peak LOD	Number of Markers	PVE (%)
*HSPA4*	eqtl_HSPA4_7	7	59.305-59.559	Marker13973	5.73	1	11.4
*HSPA5-08834*	eqtl_08834_1	1	37.775-38.056	Marker10740	6.85	3	11
	eqtl_08834_2	2	55.886-56.171	Marker27538	6.8	3	12.2
	eqtl_08834_3	3	70.226-70.319	Marker7956	7.55	1	13.7
	eqtl_08834_6	6	45.588-47.109	Marker10629	4.25	3	6
	eqtl_08834_10	10	55.184-63.734	Marker8139	3.97	14	6.6
*HSPA12A*	eqtl_HSPA12A_2	2	76.025-76.531	Marker18003	6.55	2	12.5
	eqtl_HSPA12A_6	6	85.9-86.9	Marker25714	7.12	2	13.4
*HSPA12B-22078*	eqtl_22078_2	2	75.66	Marker2650	8.52	1	17.1
	eqtl_22078_6	6	86.854	Marker25714	9.29	1	19
*HYOU1*	eqtl_HYOU1_8	8	70.508-71.201	Marker27468	7.19	4	17.2
*HSPA13*	eqtl_HSPA13_2	2	75.66	Marker2650	6.89	1	15.2
	eqtl_HSPA13_6-1	6	85.852-86.854	Marker28123	9.56	5	21.9
	eqtl_HSPA13_6-2	6	93.738-97.367	Marker36658	6.9	5	16.5
	eqtl_HSPA13_6-3	6	80.218-80.894	Marker36719	6.27	2	15.3
*HSPA12B-12492*	eqtl_12492_4	4	67.045-68.122	Marker1504	7.07	1	9.7
	eqtl_12492_5	5	29.69-31.306	Marker4421	6.31	3	10.1
	eqtl_12492_6	6	86.284-86.854	Marker25714	8.24	3	13.8
*HSPA9*	eqtl_HSPA9_2	2	56.171-56.918	Marker32411	5.87	3	10.8
	eqtl_HSPA9_3	3	58.831	Marker37157	11.39	2	24.9
	eqtl_HSPA9_7	7	59.305-59.559	Marker13973	8.02	1	16.2
*HSPA5-15492*	eqtl_15492_3-1	3	55.568-62.396	Marker37157	5.68	17	14.8
	eqtl_15492_3-2	3	70.226-70.319	Marker7956	7.61	1	19.1
	eqtl_15492_4	4	25.851-26.48	Marker20355	4.43	3	9.6
	eqtl_15492_5	5	66.47-71.853	Marker45911	4.82	4	11.2
	eqtl_15492_6	6	47.913-54.293	Marker37730	4.11	5	8.2
*SIP1*	eqtl_SIP1_3	3	68.861-68.966	Marker34975	12.71	1	26.6
*HSPB1*	eqtl_HSPB1_1	1	13.509	Marker30594	6.14	1	10.6
	eqtl_HSPB1_7	7	59.305-59.559	Marker13973	5.1	1	8.6
*DJB13*	eqtl_DJB13_4-1	4	77.435-77.904	Marker5101	7.41	2	13.4
	eqtl_DJB13_4-2	4	81.469-81.772	Marker30102	7.41	2	14.8
	eqtl_DJB13_9-1	9	31.72-31.793	Marker23397	7.77	2	15.6
	eqtl_DJB13_9-2	9	26.511-30.02	Marker32635	5.93	14	12.4
	eqtl_DJB13_9-3	9	40.641-41.260	Marker6180	4.7	5	10.1
	eqtl_DJB13_9-4	9	22.587-23.134	Marker13132	4.53	3	9.8
*DNJB4*	eqtl_DNJB4_3	3	68.966	Marker34975	6.55	1	12.4
	eqtl_DNJB4_5	5	70.735	Marker38934	6.03	1	11.3
*ATG7*	eqtl_ATG7_4	4	12.875-15	Marker12663	8.32	5	8.9
	eqtl_ATG7_5	5	80.077-84.62	Marker14346	12.74	6	15.3
	eqtl_ATG7_6	6	74.921-76.921	Marker26545	10.47	1	11.9
	eqtl_ATG7_10	10	82.583-85.39	Marker17442	8.78	2	9.3
*BRKS2*	eqtl_BRKS2_3	3	67.861-68.966	Marker34975	8.2	1	17.2
	eqtl_BRKS2_8	8	50.382-50.764	Marker35461	7.87	2	16.4
*CLCN2*	eqtl_CLCN2_8	8	101.355-101.509	Marker20478	6.17	1	15.4
	eqtl_CLCN2_9	9	46.8-47.215	Marker16655	5.84	3	14.7
*GAK*	eqtl_GAK_7	7	59.305-59.559	Marker13973	6.37	1	12
*MTMR8*	eqtl_MTMR8_5	5	30.419-33.214	Marker43580	6.92	6	12.7
	eqtl_MTMR8_9	9	45.837-48.737	Marker30815	6.4	6	15.9
*RNF123*	eqtl_RNF123_5-1	5	73.043	Marker3732	9.01	1	19.7
	eqtl_RNF123_5-2	5	67.803-72.526	Marker49357	6.61	11	15.2
	eqtl_RNF123_9-1	9	0-3	Marker8801	10.71	1	20.7
	eqtl_RNF123_9-2	9	4-5.149	Marker7204	8.35	2	15.2
	eqtl_RNF123_9-3	9	8.219-8.229	Marker31542	6.84	2	14.6
	eqtl_RNF123_9-4	9	10.631-12.125	Marker44034	7.15	6	15.1
	eqtl_RNF123_9-5	9	13.521-13.73	Marker8825	6.16	4	13.4
	eqtl_RNF123_9-6	9	20.193-20.556	Marker43279	5.89	3	12.8

## Data Availability

Not applicable.

## Supplementary Materials

The following are available online at https://www.mdpi.com/article/10.3390/genes12071040/s1, Table S1: Peripheral amplification primers and unidirectional oligonucleotide primers of SNPs, Table S2: Gene expression level of 21 heat-responsive genes in 106 individuals, Figure S1: Histogram of heat-responsive gene expression distribution in the mapping family, Figure S2: Plots of eQTL mapping of 21 heat-responsive genes, Table S3: Candidate genes identified from eQTL mapping of C. gigas, Figure S3: Classification analysis of KEGG pathway of 831 key candidate genes, Figure S4: Enrichment analysis of Gene Ontology of 831 key candidate genes, Table S4: Genotype and allele frequencies of these six SNPs, Table S5: The association between SNPs and the gene expression level, Table S6: The association between SNPs and the gene expression level.
