# Supplementary figures and images for: Identification of Distant Regulatory Elements Using Expression Quantitative Trait Loci Mapping for Heat-Responsive Genes in Oysters
Source: Genes (Basel). 2021 Jul 5;12(7):1040. doi: 10.3390/genes12071040 (PMC8303352; doi:10.3390/genes12071040)

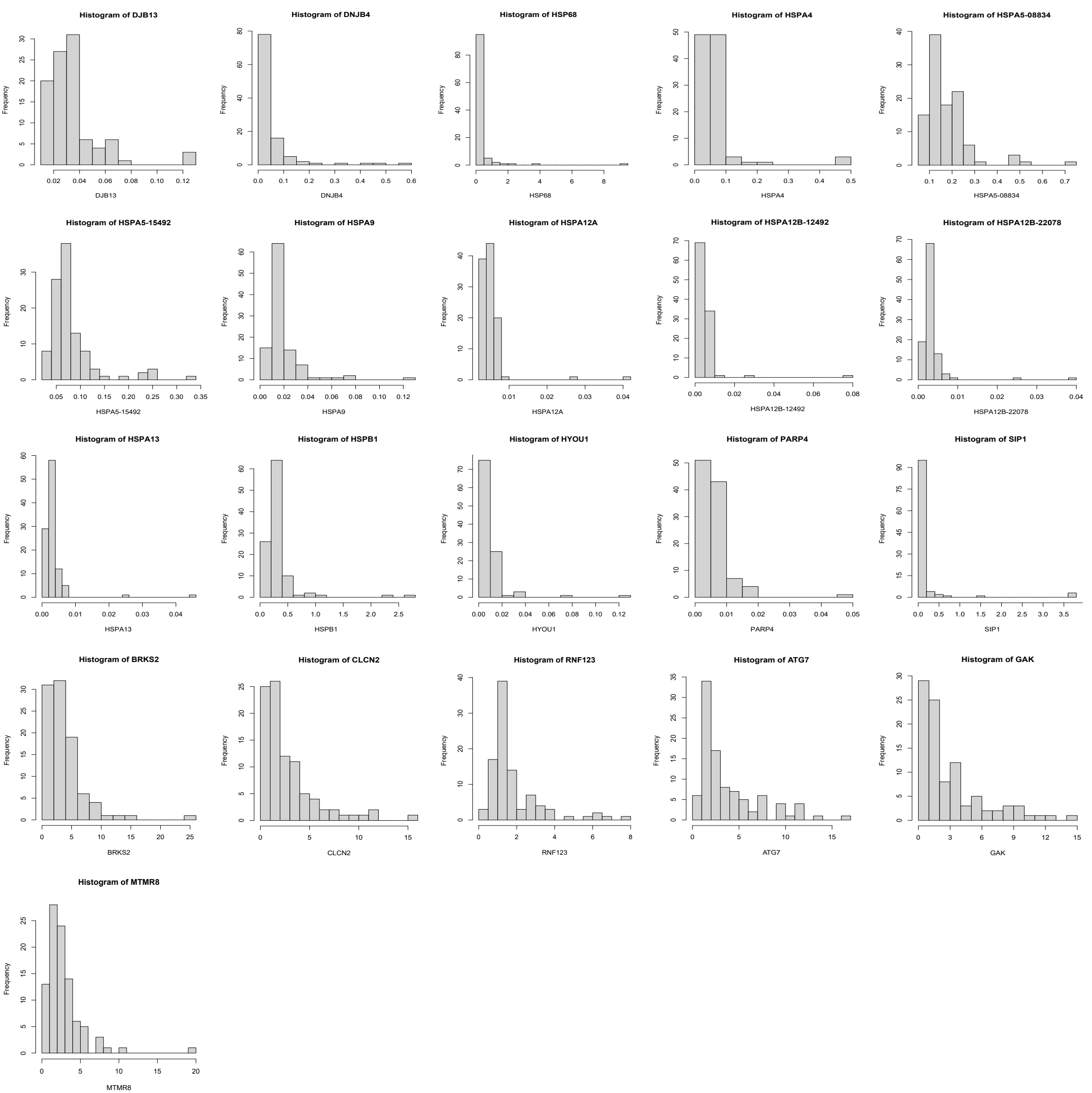

Supplement: Supplementary file 1 [file genes-12-01040-s001.zip › Figure S1.pdf]

**Figure S2.** Plots of eQTL mapping of 21 heat-responsive genes

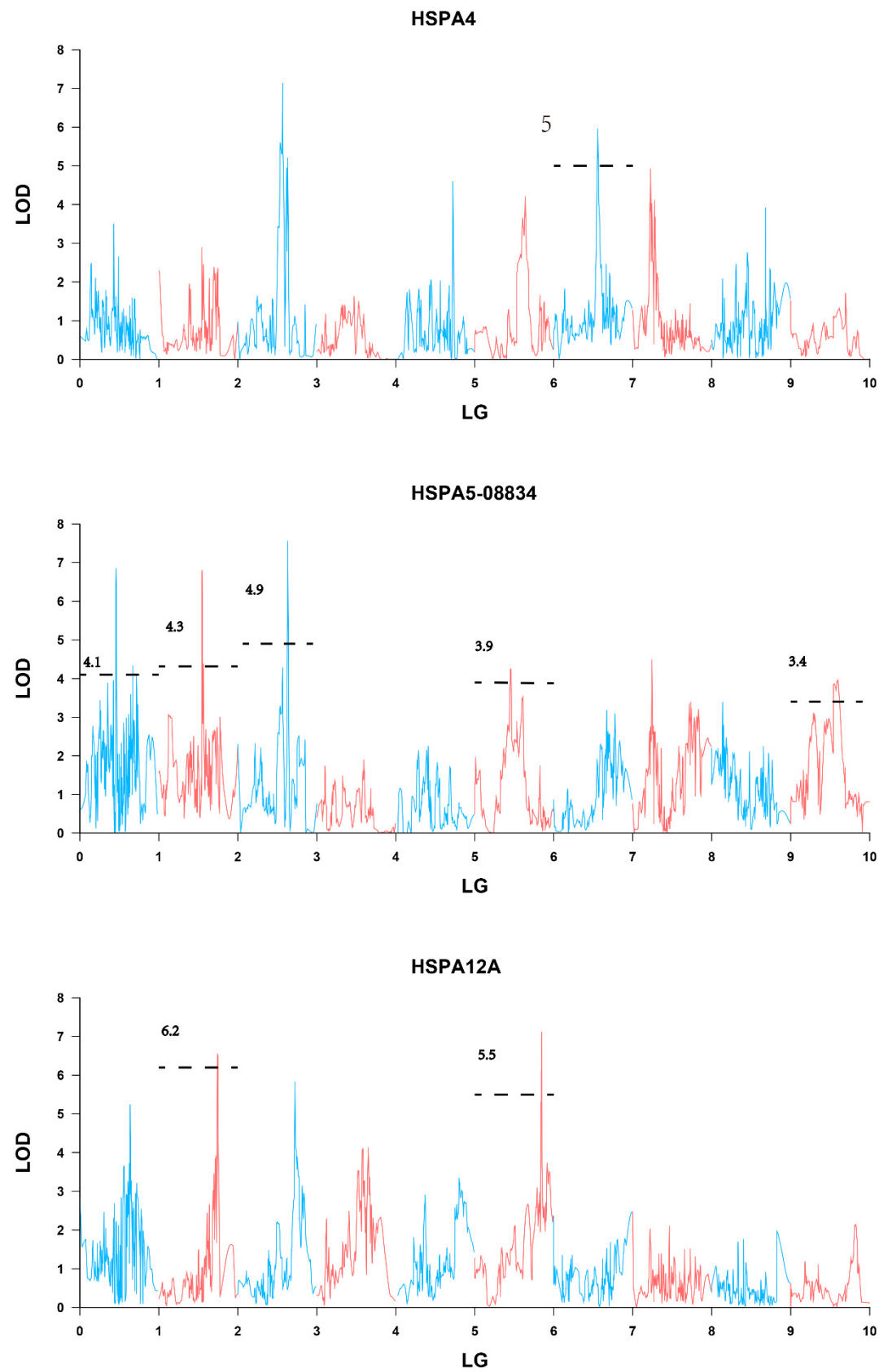

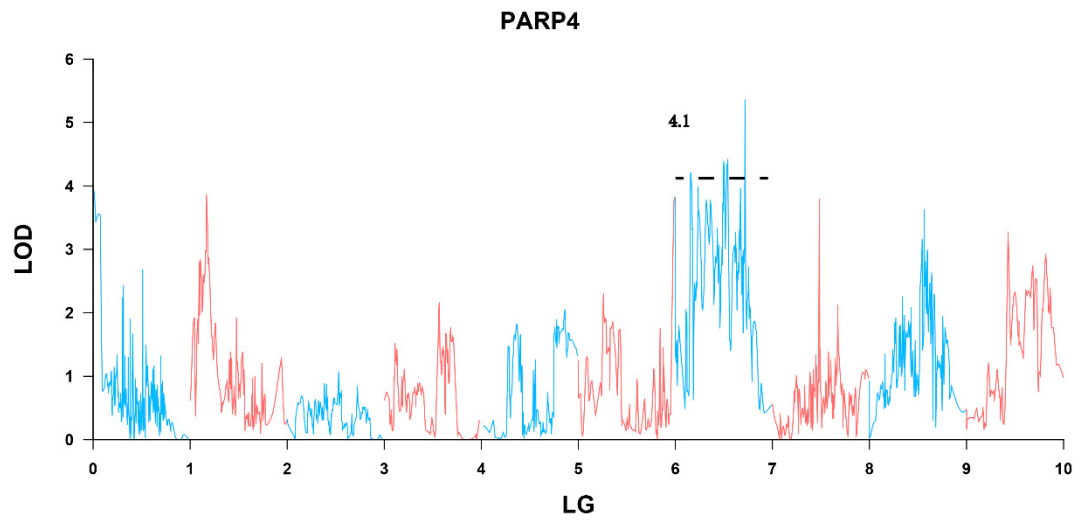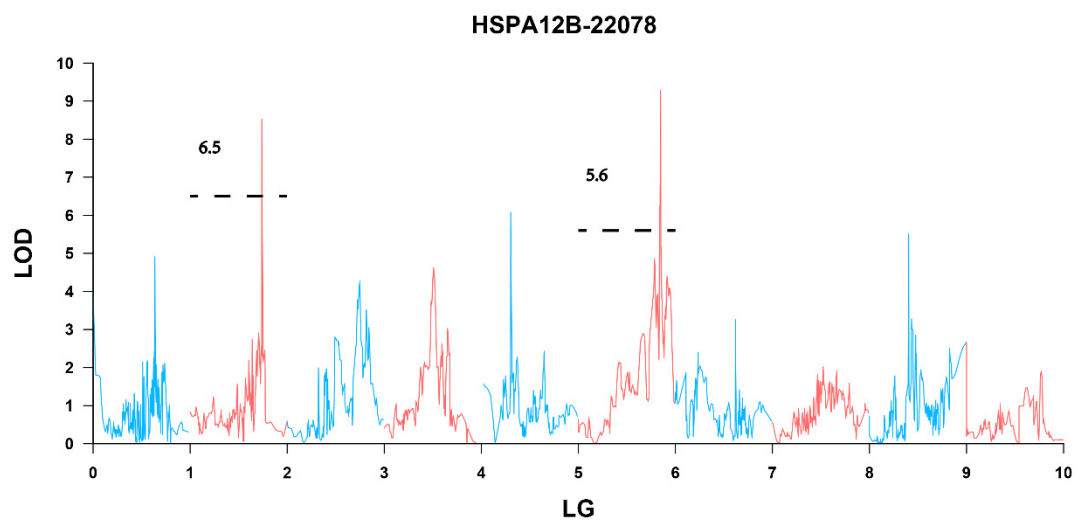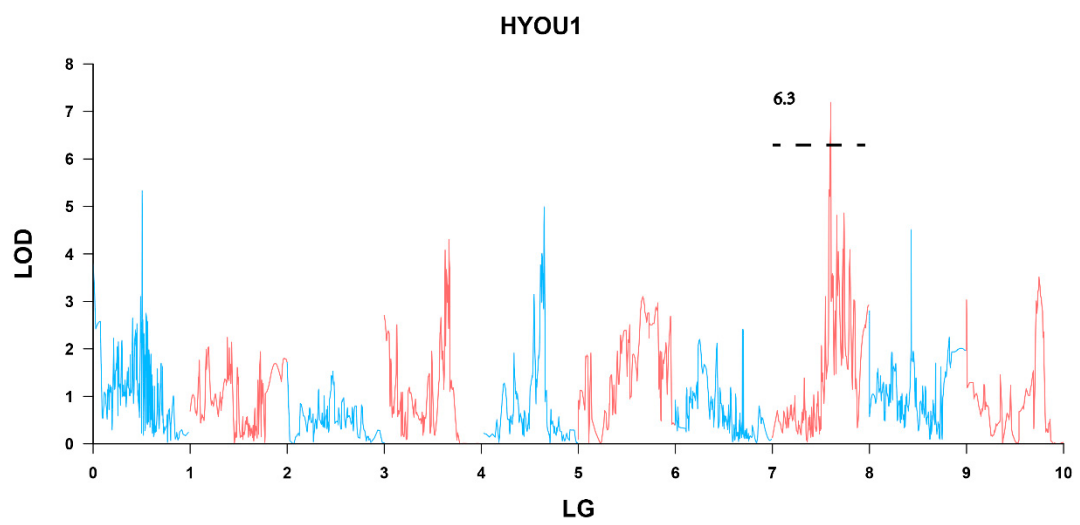

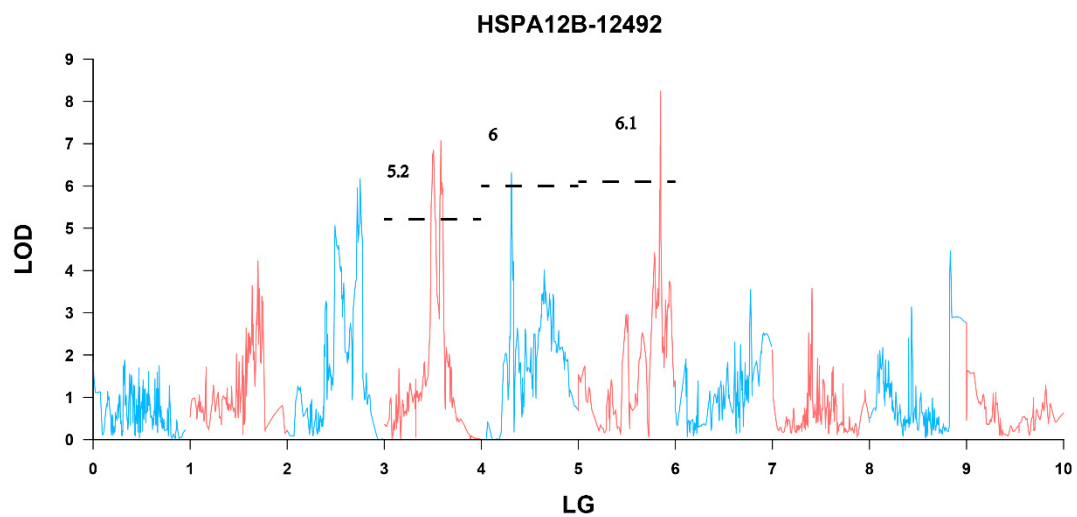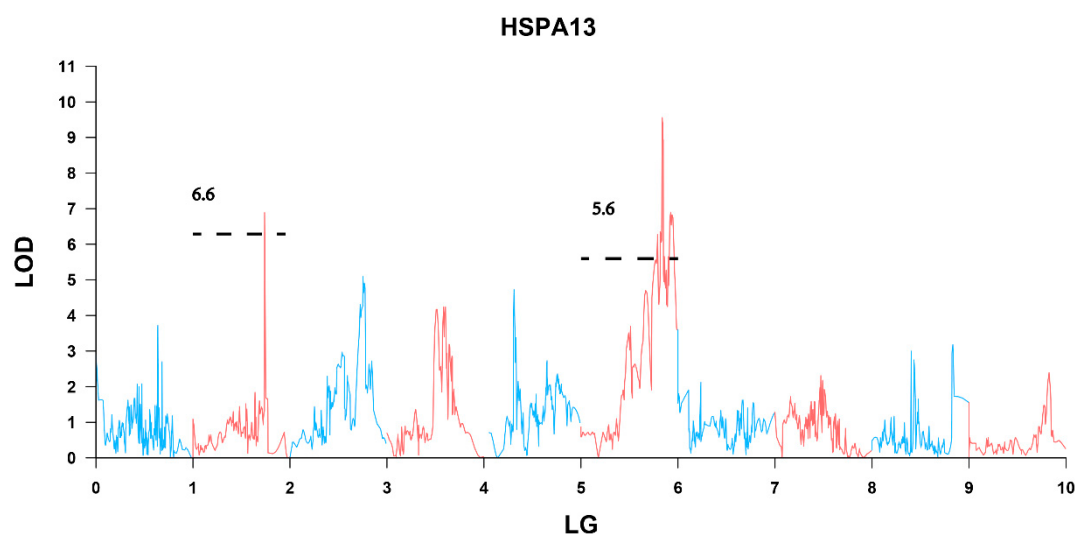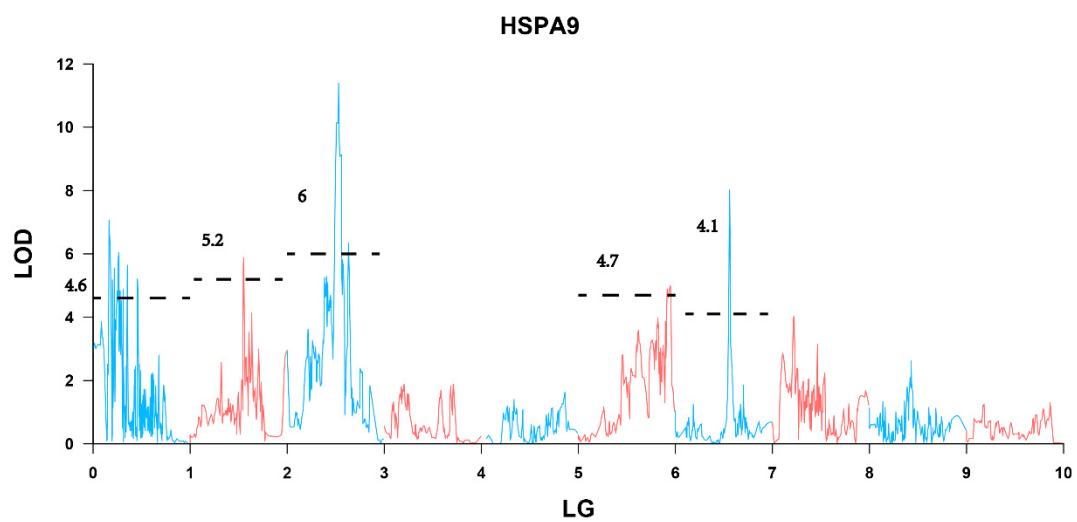

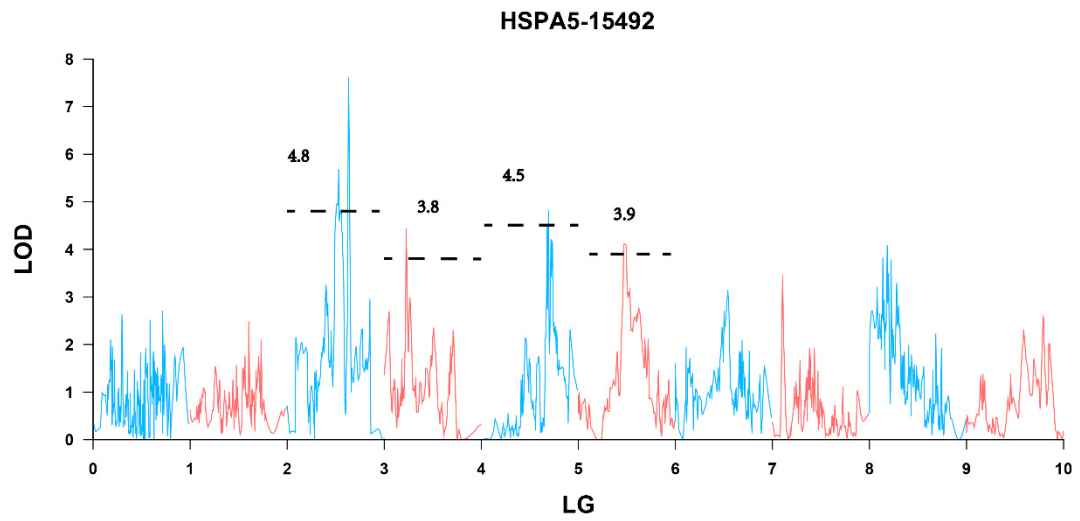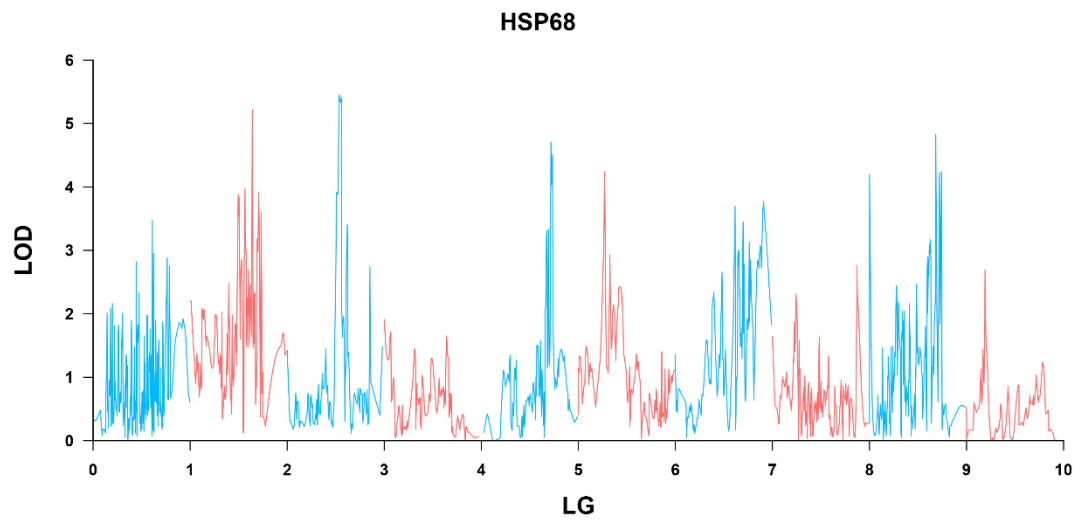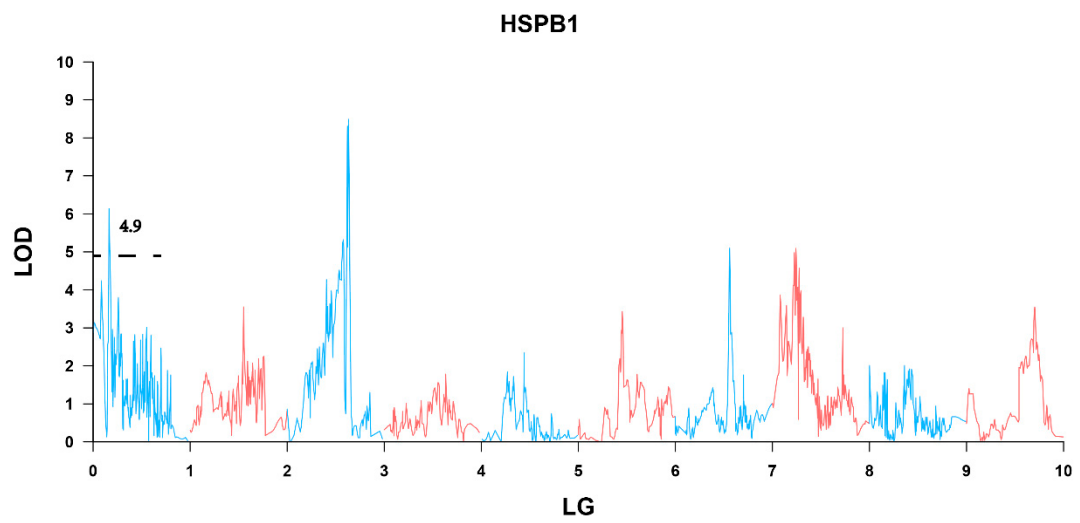

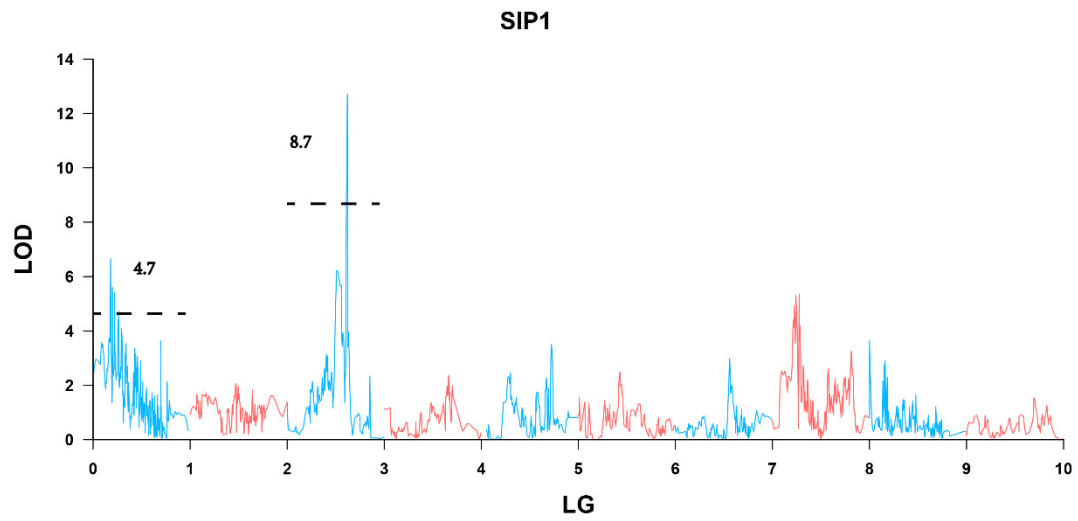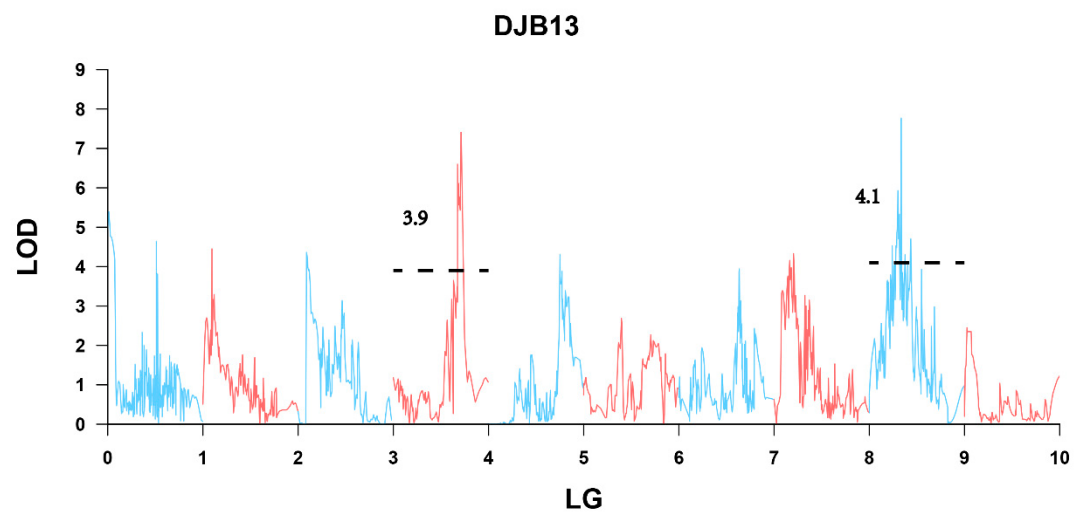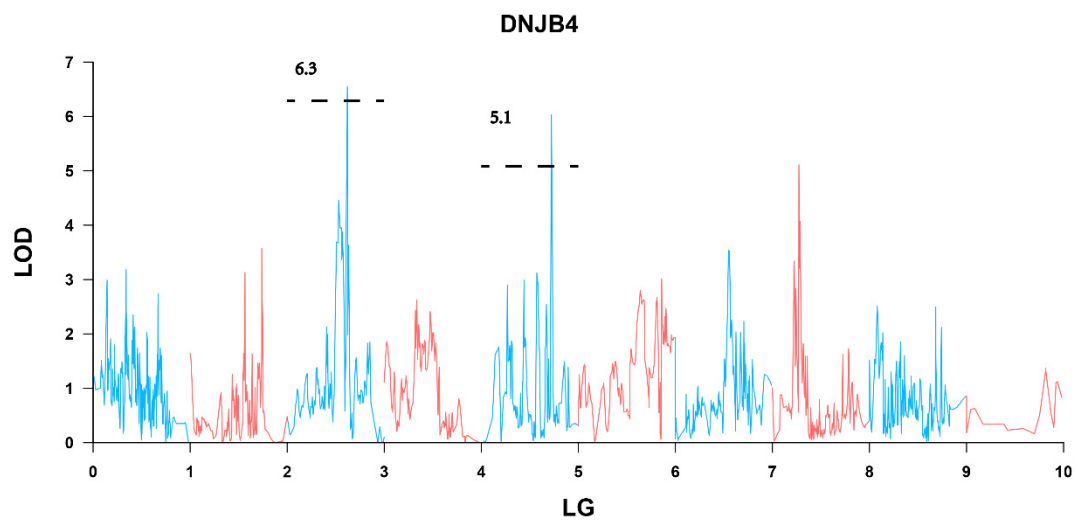

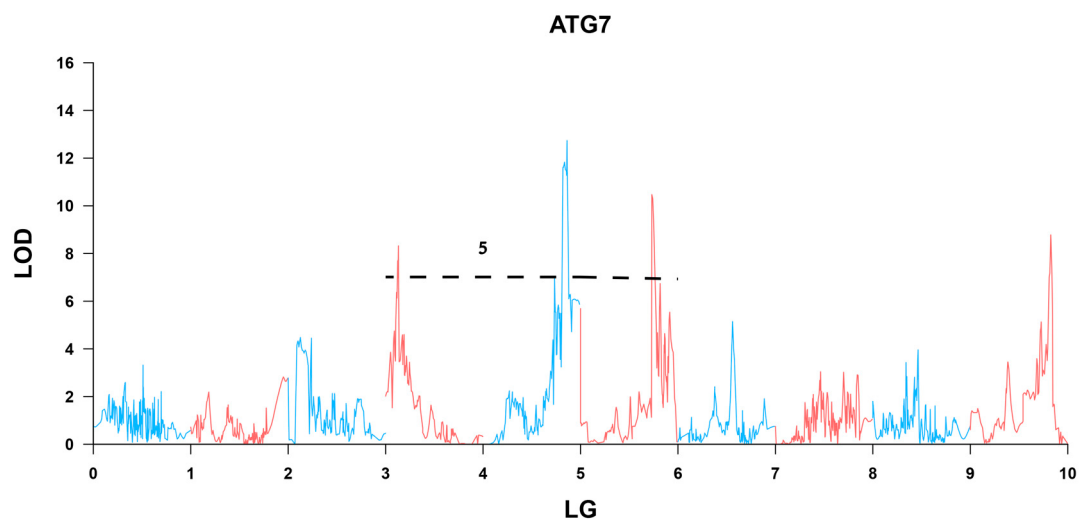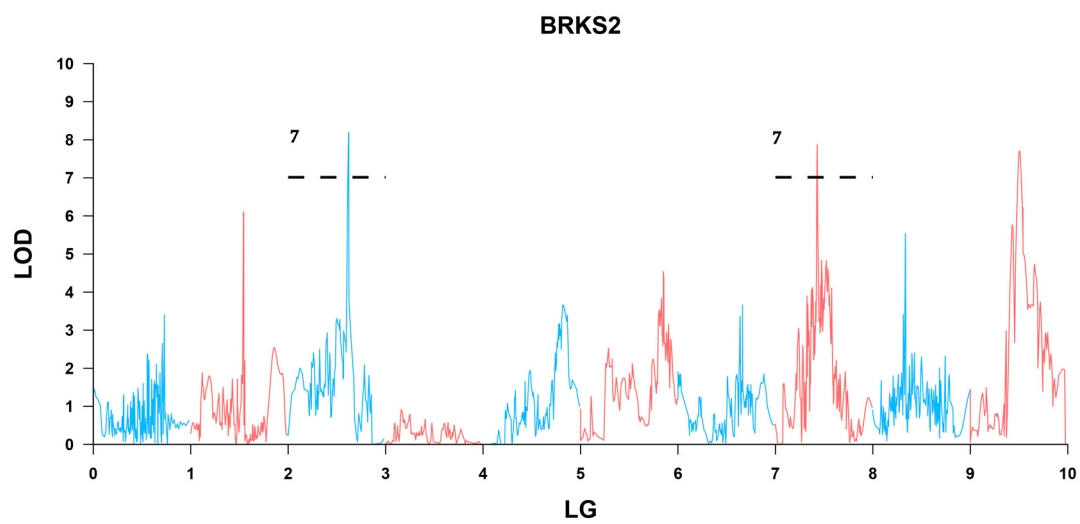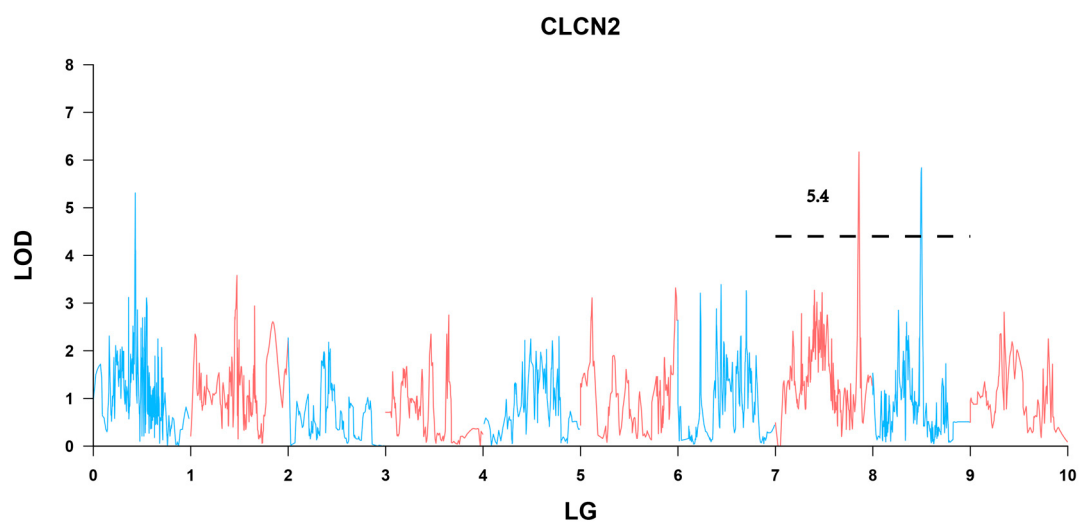

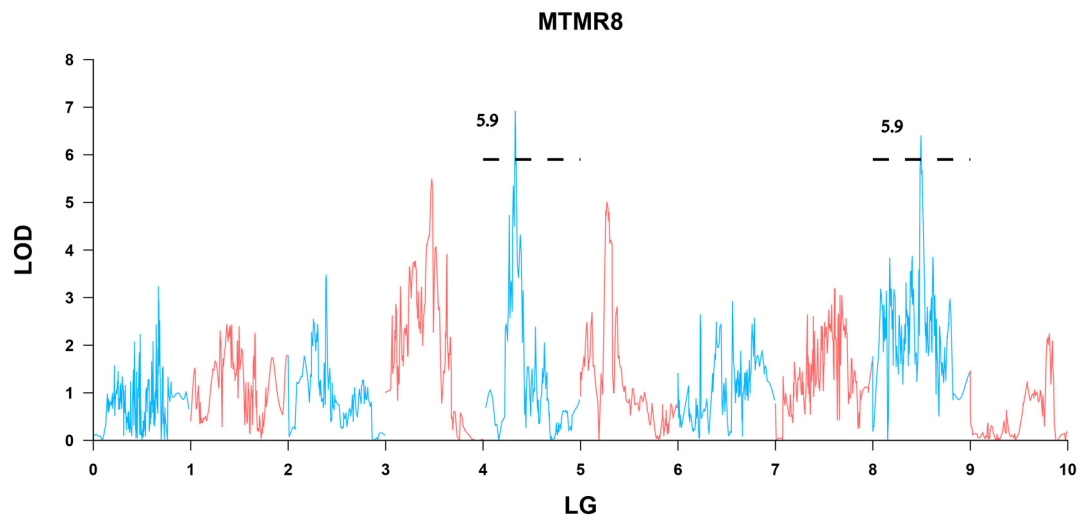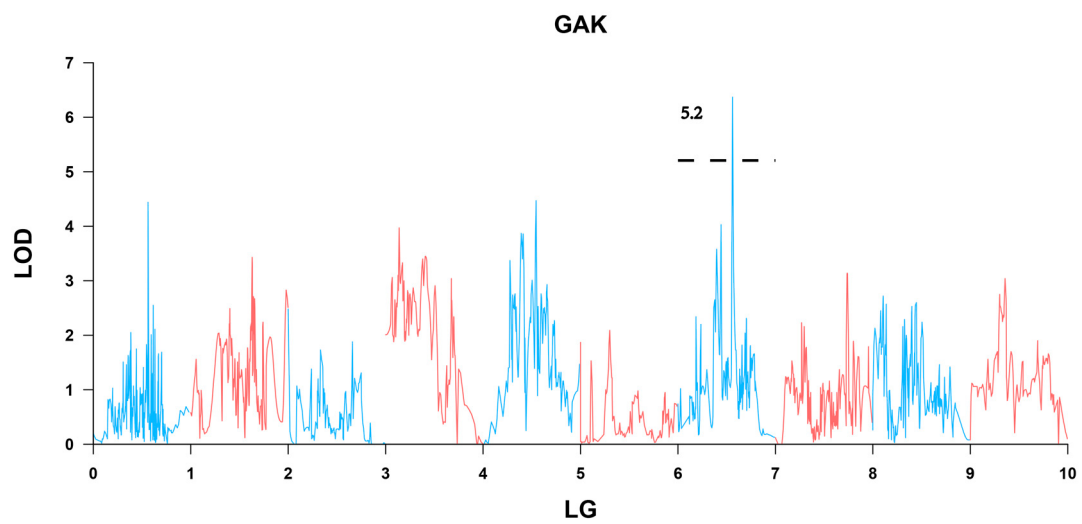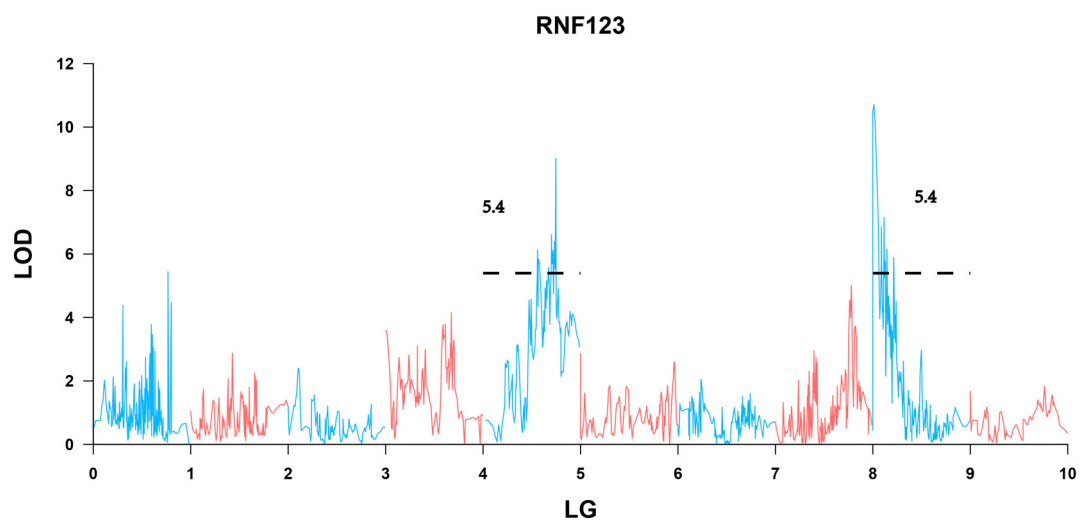

Supplement: Supplementary file 1 [file genes-12-01040-s001.zip › Figure S2.pdf]

## The Most enriched GO Terms

GO term

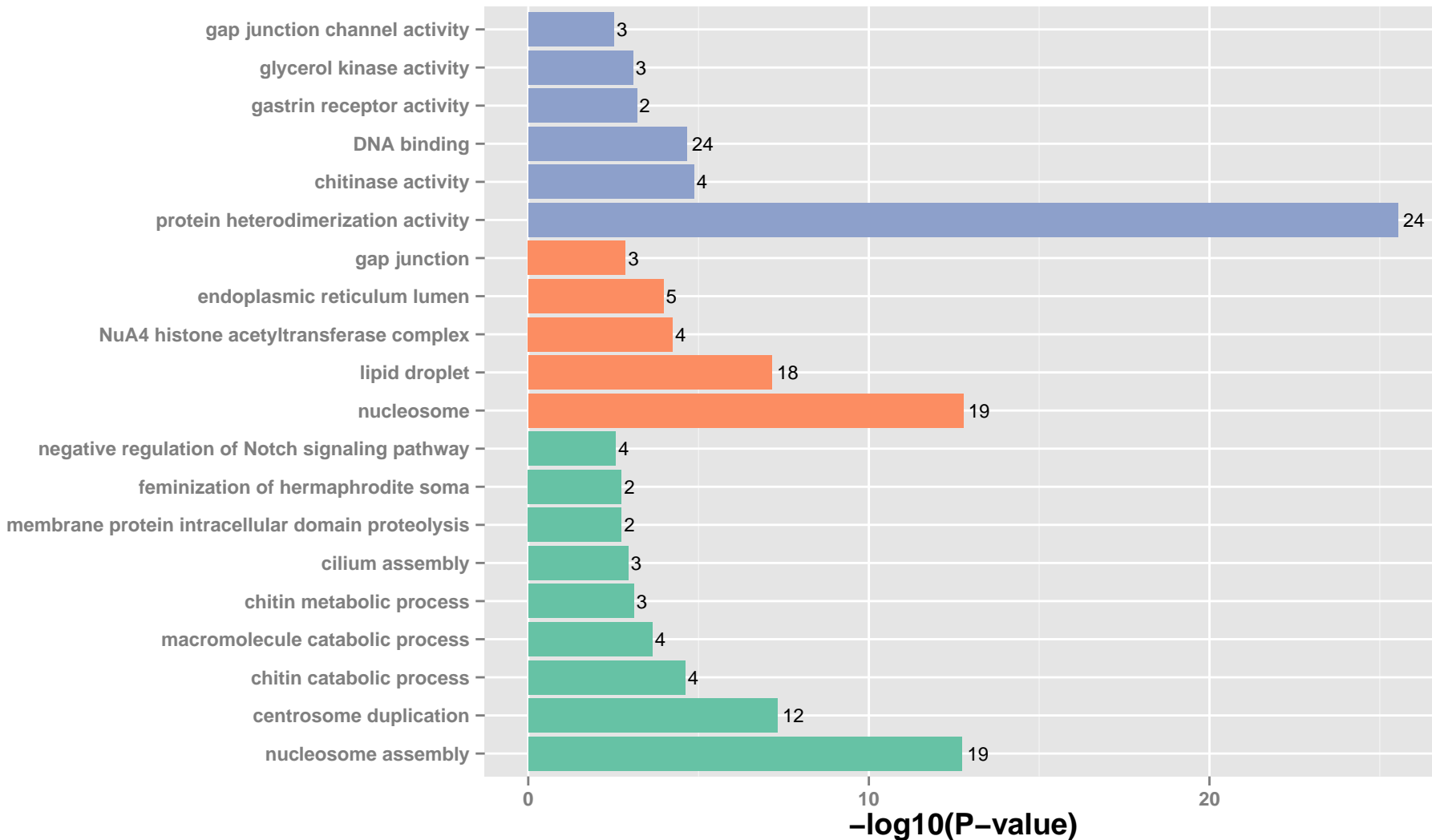

Biological Process  
Cellular Component  
Molecular Function

Supplement: Supplementary file 1 [file genes-12-01040-s001.zip › Figure S4.pdf]
